# Prognostic value of the neutrophil percentage-to-albumin ratio for all-cause and cardiovascular disease mortality in individuals with coronary heart disease: A cohort study

**DOI:** 10.1097/MD.0000000000046154

**Published:** 2025-11-28

**Authors:** Han Yan, Jiayi Chen, Haorui Zha, Linghua Pei

**Affiliations:** a The Second Clinical Medical College, Zhejiang Chinese Medical University, Hangzhou, China; b Department of Cardiology, The Second Affiliated Hospital of Zhejiang Chinese Medicine University, Hangzhou, China.

**Keywords:** coronary heart disease, mortality, neutrophil percentage-to-albumin ratio, NHANES

## Abstract

The association between the neutrophil percentage-to-albumin ratio (NPAR) and all-cause and cardiovascular disease mortality in individuals with coronary heart disease (CHD) remains unclear. This study aimed to investigate the correlation between NPAR levels and the risks of all-cause and cardiovascular disease mortality within this specific patient population. This research included 935 participants diagnosed with CHD. We conducted survival analyses using the Kaplan–Meier method and employed multivariate Cox proportional hazards regression models to assess the association between NPAR, categorized into tertiles, and the risk of all-cause and cardiovascular mortality. Additionally, we utilized restricted cubic splines to analyze potential nonlinear relationships and performed subgroup analyses based on relevant covariates to identify populations potentially at higher risk. Over the follow-up period, 260 all-cause deaths and 100 cardiovascular deaths were recorded among the participants. Multivariate Cox regression analysis demonstrated that, compared to the lowest NPAR tertile (*Q*1), participants in the highest tertile (*Q*3) had significantly elevated risks of all-cause mortality (hazard ratio = 1.76, 95% confidence interval: 1.19–2.61, *P* = .005) and cardiovascular mortality (hazard ratio = 3.12, 95% confidence interval: 1.67–5.81, *P* < .001). The restricted cubic splines analysis confirmed a nonlinear association between continuous NPAR levels and both all-cause and cardiovascular mortality. Higher NPAR levels were significantly associated with an increased likelihood of both all-cause and cardiovascular mortality among patients with CHD. These findings suggest that NPAR could serve as a potentially valuable prognostic marker for predicting mortality risk in individuals affected by CHD.

## 1. Introduction

Coronary heart disease (CHD) is a significant threat to public health globally. According to 2017 data, cardiovascular disease (CVD) deaths reached 17.79 million globally,^[1]^ with ischemic heart disease responsible for nearly half of these.^[2]^ In the United States (US), over 350,000 deaths occur annually due to CHD.^[3]^ Despite notable advancements in managing this condition, mortality and disability rates remain high, ranking CHD among the leading causes of morbidity and mortality worldwide.

Chronic inflammation is acknowledged as a significant risk factor for CVD. Inflammatory cell infiltration in the infarct zone is a key element in the pathogenesis of CHD, and the degree of infiltration is closely related to prognosis.^[4]^ Neutrophils are key players in the inflammatory process, serving as central mediators in the body’s inflammatory response. Their levels are recognized as crucial indicators for both acute and chronic inflammatory conditions. Serum albumin (ALB) is the major plasma protein and a key indicator for clinical diagnosis, as well as metabolic and nutritional status assessment. Decreased ALB levels (hypoalbuminemia) are associated with inflammation, infection, and malnutrition. Studies have shown an association between ALB and cardiovascular disease, as albumin exhibits protective effects against inflammation and oxidative stress on vascular endothelial function^[5]^ and inhibits platelet-activating factors, counteracting platelet aggregation.^[6]^

The neutrophil percentage-to-albumin ratio (NPAR), combining these 2 clinical markers, represents a comprehensive biomarker.^[7]^ It has been used to assess the prognosis of various diseases, including nonalcoholic fatty liver disease, advanced liver fibrosis,^[8]^ stroke,^[1]^ cancer,^[9]^ and CVD.^[10]^

Although NPAR has emerged as a potentially reliable indicator for predicting mortality in various inflammatory diseases, relatively few studies have specifically explored its utility for predicting outcomes in individuals with CHD. Therefore, this study aimed to analyze the association of NPAR with cardiovascular and all-cause mortality in patients with CHD using data from the National Health and Nutrition Examination Survey (NHANES) database.

## 2. Materials and methods

### 2.1. Study design

NHANES is a comprehensive and emblematic study that uses a complex sampling approach to gather information on the health and nutritional conditions of households across the United State. This extensive dataset includes insights on demographics, dietary habits and examination data from the laboratory. We collected information on participants interviewed between 2009 and 2018. Additionally, a longitudinal cohort was developed by connecting it with the National Death Index from the National Center for Health Statistics to track participant survival status. The study was approved by the National Center for Health Statistics Research Ethics Review Board, and all participants provided written informed consent.

### 2.2. Study population

**Figure 1.**
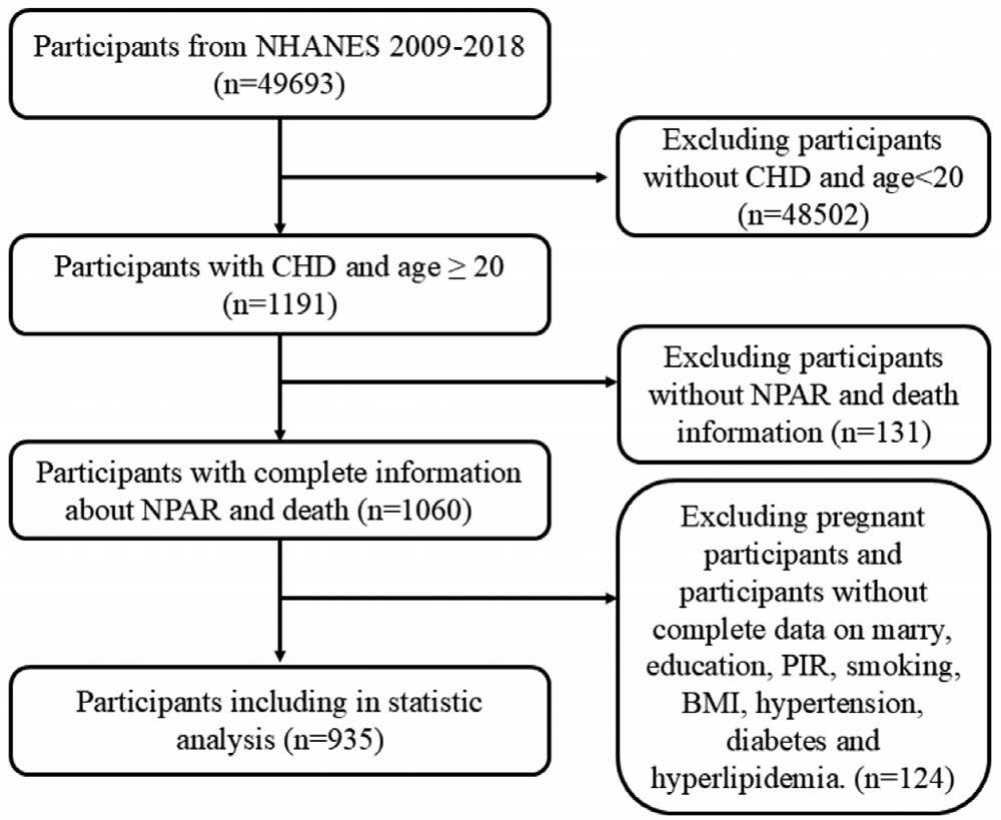
Participant screening flowchart. BMI = body mass index, CHD = coronary heart disease, NHANES = National Health and Nutrition Examination Survey, NPAR = neutrophil percentage-to-albumin ratio, PIR = poverty-to-income ratio.

This research utilized data from the NHANES 2009 to 2018 cycles. CHD status was ascertained via self-report; participants were asked if a doctor or health professional had ever told them they had CHD. Participants responding affirmatively were classified as having CHD. From the initial pool of participants across these cycles, we excluded those aged <20 years, those with missing data for ALB or neutrophil percentage (N%), those missing mortality follow-up data, pregnant individuals, and those with missing data for covariates included in the final models. After applying these exclusion criteria, a total of 935 participants with CHD were included in the final analysis (Fig. 1).

### 2.3. Study variables

#### 2.3.1. Determination of mortality

The primary outcomes of this study were all-cause and CVD mortality. To ascertain the survival outcomes of the study cohort, we used death files linked from the 2009–2018 NHANES database, with participants monitored up until December 31, 2019. All-cause mortality was categorized as any deceased cause noted in the records. CVD mortality was defined as death resulting from cardiovascular causes, identified using the international classification of diseases, tenth revision codes I00-I09, I11, I13, and I20-I51. This approach allows for the examination of a broad range of fatal cardiovascular events in this specific high-risk cohort of individuals with preexisting CHD, providing a comprehensive view of their long-term prognosis.

#### 2.3.2. Definition of NPAR

NPAR is derived from blood samples, which are processed and sent by professionals to a designated location for assaying. For a comprehensive rundown of the lab procedures, check out the NHANES website. The NPAR was computed using the equation: N (%)/ALB (g/dL). We categorized the participants into 3 groups (*Q*1–*Q*3) based on their NPAR values according to tertiles, the *Q*1 group served as the benchmark for comparison.

#### 2.3.3. Assessment of covariates

The study included a range of covariates that could potentially influence the results, including: age, gender, ethnicity, marry, education level, poverty-to-income ratio (PIR), smoking status, body mass index (BMI), hypertension, diabetes, hyperlipidemia, serum glucose, glycated hemoglobin, triglycerides, total cholesterol, low-density lipoprotein, and high-density lipoprotein. Among these, ethnicity is divided into Mexican American, other Hispanic, non-Hispanic White, non-Hispanic Black, Other Race. Marry is divided into never married, widowed, divorced or separated and married status or cohabiting. Educational levels are divided into 3 categories: less than high school, high school or equivalent, college or above. Smoking status was determined based on responses “whether they smoked at least 100 cigarettes in life” and “Do you now smoke cigarettes?” was divided into 3 categories: never, former and now. According to the 2017 American Heart Association Recommended Guidelines,^[11]^ hypertension was defined as an average systolic blood pressure ≥ 130 mm Hg and/or diastolic blood pressure (DBP) ≥ 80 mm Hg based on three readings taken in a quiet state; self-reported use of oral hypertension medication; or a confirmed diagnosis by a medical professional. To be classified with diabetes, an individual must meet one of these criteria^[12]^: a medical professional or physician has diagnosed it; taking medications to lower blood sugar or insulin; fasting blood glucose ≥ 7.0 mmol/L; glycated hemoglobin ≥ 6.5%. Hyperlipidemia is defined as a total cholesterol level of ≥200 mg/dL, a triglycerides level of ≥150 mg/dL, low-density lipoprotein level of ≥130 mg/dL, or high-density lipoprotein level of ≥50 mg/dL in women and ≥40 mg/dL in men, or people who had used cholesterol-lowering drugs were also classified as hyperlipidemic. Blood sample data were obtained through experimental data in NHANES.

### 2.4. Statistical analysis

Given the intricate nature of the NHANES sampling framework, we integrated sample weights, clustering and stratification into our analytical procedures. Categorical data were presented as frequencies or percentages, and continuous data were represented as median values and inter-quartile spacing. To evaluate inter-group disparities in continuous variables, we employed the Kruskal–Wallis test, and for categorical variables, we utilized the chi-squared test. We used Kaplan–Meier curves and their log-rank tests to assess cumulative survival differences between NPAR outcomes. The study evaluated the link between NPAR and overall mortality and CVD mortality using a multivariate Cox regression model. The findings were presented as hazard ratios (HR) along with their corresponding 95% confidence intervals (CI). Three distinct models were developed: model 1: no adjustment for confounders. Model 2: factored in age, gender and ethnicity. Model 3: additionally, PIR, marry, education, BMI, smoking, hypertension, hyperlipidemia and diabetes were added to model 2. Moreover, we applied a restricted cubic splines model to delve into the connections between NPAR and mortality from all causes and CVD. We also carried out subgroup analyses focusing on age, gender, ethnicity, PIR, marry, education, BMI, smoking, hypertension, hyperlipidemia and diabetes to explore their impact on the results.

All statistical analyses were performed using R (Version 4.2.0), with 2-tailed tests and a significance level set at *P* < .05.

## 3. Results

### 3.1. Baseline population characteristics stratified by NPAR

The study included 935 participants with CHD (median age 70 years, 67.6% male). Participants were divided into tertiles based on their NPAR values (*Q*1: NPAR ≤ 13.49, *Q*2: NPAR ≤ 15.83, *Q*3: NPAR > 15.83). Significant differences in baseline characteristics were observed across NPAR tertiles, particularly for age, ethnicity, diabetes prevalence, smoking status, and DBP (all *P* < .05). Notably, participants in the highest NPAR tertile (*Q*3) were significantly older, had a higher prevalence of diabetes, and lower DBP compared to those in the lowest tertile (*Q*1). Full details are presented in Table 1.

**Table 1:** Baseline characteristics of participants.

Characteristics	Overall (n = 935)	*Q*1	*Q*2	*Q*3	*P*-value
		(NPAR ≤ 13.49, n = 312)	(NPAR ≤ 15.83, n = 311)	(NPAR > 15.83, n = 312)	
Age, yr	70.00 (17.00)	68.00 (16.00)	71.00 (16.50)	72.50 (16.00)	**<.001**
Gender					.157
Male	632 (67.6)	216 (69.2)	218 (70.1)	198 (63.5)	
Female	303 (32.4)	96 (30.8)	93 (29.9)	114 (36.5)	
Ethnicity					**<.001**
Mexican America	76 (8.1)	28 (9.0)	25 (8.0)	23 (7.4)	
Other Hispanic	75 (8.0)	25 (8.0)	26 (8.4)	24 (7.7)	
Non-Hispanic White	581 (62.1)	167 (53.5)	197 (63.3)	217 (69.6)	
Non-Hispanic Black	129 (13.8)	67 (21.5)	33 (10.6)	29 (9.3)	
Other	74 (7.9)	25 (8.0)	30 (9.6)	19 (6.1)	
Marry					.448
Never married	55 (5.9)	18 (5.8)	21 (6.8)	16 (5.1)	
Widowed, divorced or separated	320 (34.2)	103 (33.0)	98 (31.5)	119 (38.1)	
Married status or cohabiting	560 (59.9)	191 (61.2)	192 (61.7)	177 (56.7)	
Education					.168
Less than high school	272 (29.1)	93 (29.8)	97 (31.2)	82 (26.3)	
High school or equivalent	218 (23.3)	60 (19.2)	75 (24.1)	83 (26.6)	
College or above	445 (47.6)	159 (51.0)	139 (44.7)	147 (47.1)	
PIR	1.85 (2.46)	1.87 (2.69)	1.81 (2.45)	1.85 (2.30)	.869
Smoking					.047
Never	359 (38.4)	139 (44.6)	118 (37.9)	102 (32.7)	
Former	412 (44.1)	121 (38.8)	139 (44.7)	152 (48.7)	
Now	164 (17.5)	52 (16.7)	54 (17.4)	58 (18.6)	
BMI	29.30 (7.95)	28.95 (7.14)	29.20 (8.18)	29.80 (8.73)	.202
Hypertension					.572
No	140 (15.0)	44 (14.1)	52 (16.7)	44 (14.1)	
Yes	795 (85.0)	268 (85.9)	259 (83.3)	268 (85.9)	
Diabetes					**<.001**
No	523 (55.9)	200 (64.1)	173 (55.6)	150 (48.1)	
Yes	412 (44.1)	112 (35.9)	138 (44.4)	162 (51.9)	
Hyperlipidemia					.368
No	214 (22.9)	63 (20.2)	74 (23.8)	77 (24.7)	
Yes	721 (77.1)	249 (79.8)	237 (76.2)	235 (75.3)	
SBP (mm Hg)	129.00 (25.33)	128.67 (25.00)	128.67 (25.33)	129.33 (24.00)	.944
DBP (mm Hg)	66.00 (16.67)	67.33 (15.50)	65.33 (17.33)	64.00 (17.33)	.001
HDL (mg/dL)	46.00 (18.00)	45.00 (19.00)	45.00 (17.00)	46.50 (18.25)	.273
LDL (mg/dl)	87.00 (44.50)	89.00 (48.00)	89.00 (45.50)	82.00 (43.00)	.259
TG (mg/dL)	108.50 (77.50)	104.50 (95.75)	104.50 (77.00)	105.00 (65.50)	.349
TC (mg/dL)	165.00 (54.00)	166.00 (55.00)	166 (62.00)	161.00 (48.25)	.086

Data were presented as median (IQR) or N (%). Values in bold indicate statistical significance (*P* < .001).

DBP = diastolic blood pressure, HDL = high-density lipoprotein, LDL = low-density lipoprotein, PIR = poverty-to-income ratio, SBP = systolic blood pressure, TC = total cholesterol, TG = triglycerides.

### 3.2. The relationship between NPAR and mortality

Throughout the subsequent monitoring phase of this investigation, we documented 260 fatalities due to any cause and 100 deaths specifically from CVD (Table 2). We evaluated the association between NPAR tertiles and mortality using 3 nested Cox proportional hazards models. Compared to the lowest NPAR tertile (*Q*1, reference group), higher NPAR tertiles were associated with increased mortality risk. In the fully adjusted model (model 3, adjusting for age, gender, ethnicity, PIR, marry, education level, BMI, smoking status, hypertension, hyperlipidemia, and diabetes), participants in the highest tertile (*Q*3) had a significantly higher risk of all-cause mortality (HR = 1.76, 95% CI: 1.19–2.61, *P* = .005) and CVD mortality (HR = 3.12, 95% CI: 1.67–5.81, *P* < .001) compared to those in the *Q*1 group. Significant positive trends were observed across increasing NPAR tertiles for both all-cause mortality (*P* for trend = .002) and CVD mortality (*P* for trend < .001).

**Table 2:** Association between NPAR and all-cause and CVD mortality rates.

	*Q*1	*P*-value	*Q*2	*P*-value	*Q*3	*P*-value	*P*-trend
All cause mortality							
Death population	61		81		118		
Model 1	1	Reference	1.50 (0.98, 2.28)	.06	2.67 (1.78, 3.99)	<.001	<.001
Model 2	1	Reference	1.26 (0.83, 1.91)	.271	2.08 (1.39, 3.11)	<.001	<.001
Model 3	1	Reference	1.13 (0.72, 1.77)	.607	1.76 (1.19, 2.61)	.005	.002
CVD mortality							
Death population	19		31		50		
Model 1	1	Reference	2.44 (1.13, 5.25)	.023	4.44 (2.35, 8.39)	<.001	<.001
Model 2	1	Reference	2.02 (0.93, 4.37)	.075	3.48 (1.81, 6.66)	<.001	<.001
Model 3	1	Reference	1.90 (0.87, 4.17)	.11	3.12 (1.67, 5.81)	<.001	<.001

Model 1 was the crude model.

Model 2 was adjusted by age, gender and ethnicity.

Model 3 was adjusted by age, gender, ethnicity, PIR, marry, education, BMI, smoking, hypertension, hyperlipidemia, and diabetes.

CVD = cardiovascular disease, NPAR = neutrophil percentage-to-albumin ratio.

In the unadjusted model (model 1), the association was stronger (*Q*3 vs *Q*1: HR = 2.67, 95% CI: 1.78–3.99 for all-cause; HR = 4.44, 95% CI: 2.35–8.39 for CVD mortality, both *P* < .001). The association remained significant but was attenuated after adjusting for age, gender, and ethnicity (model 2). These results indicate that elevated NPAR is an independent predictor of increased all-cause and CVD mortality risk in individuals with CHD, even after controlling for a wide range of potential confounders.

### 3.3. Nonlinear detection of NPAR and mortality rates

**Figure 2.**
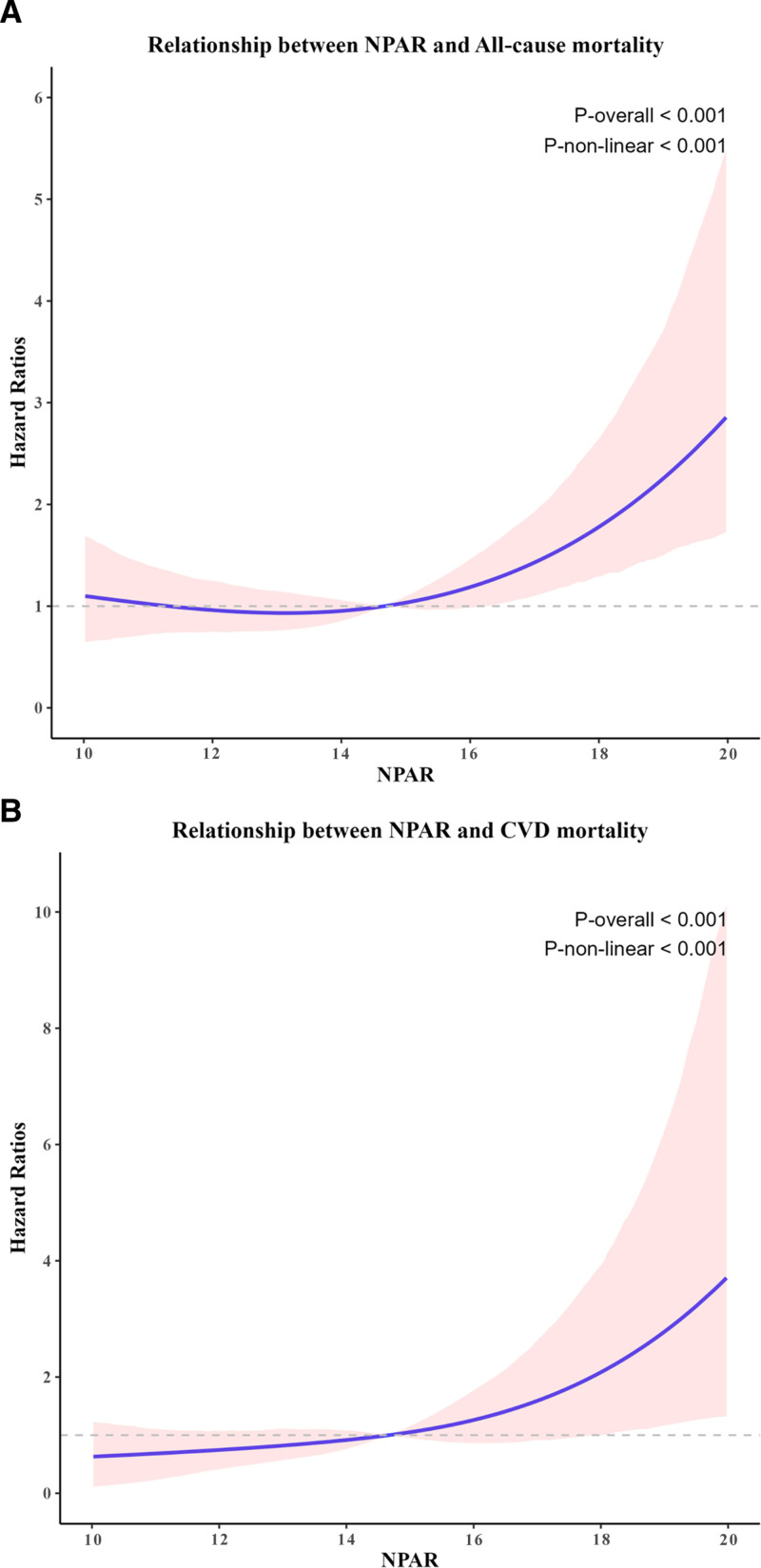
Association of NPAR with all-cause (A) and CVD (B) mortality. CVD = cardiovascular disease, NPAR = neutrophil percentage-to-albumin ratio.

Using restricted cubic splines in the fully adjusted Cox model, we found evidence of a significant nonlinear relationship between continuous NPAR levels and both all-cause mortality (*P* for nonlinearity < .001) and CVD mortality (*P* for nonlinearity < .001; Fig. 2). The risk of both mortality outcomes remained relatively stable at lower NPAR levels but increased sharply above an NPAR value of approximately 16. The HRs, relative to the median NPAR value, progressively increased at higher NPAR levels.

### 3.4. Kaplan–Meier survival analysis

**Figure 3.**
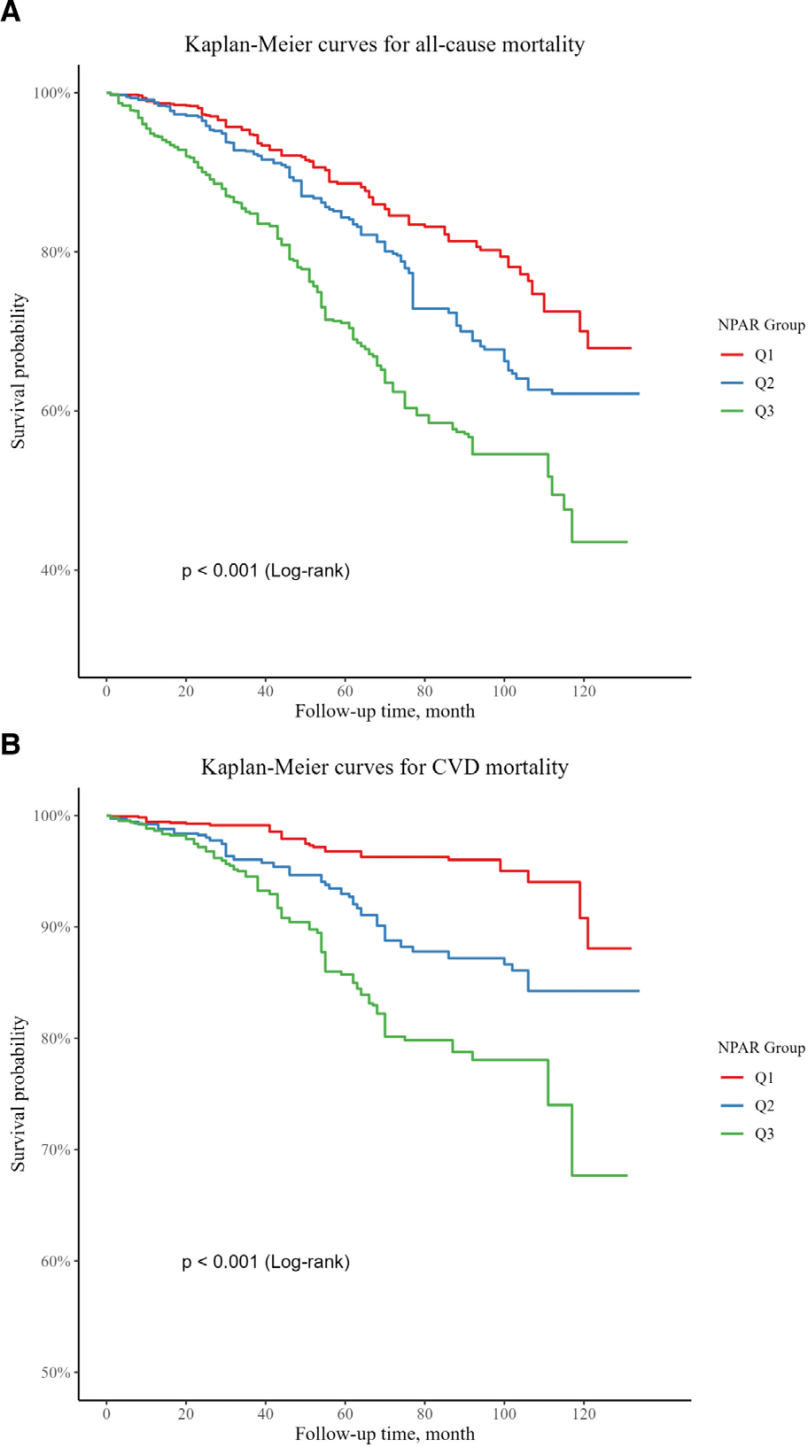
Kaplan–Meier survival curves for all-cause (A) and CVD (B) mortality. CVD = cardiovascular disease.

Kaplan–Meier survival curves (Fig. 3) demonstrated significantly lower survival probabilities for both all-cause and CVD mortality among participants in the higher NPAR tertiles (*Q*2 and *Q*3) compared to the lowest tertile (*Q*1). The log-rank test indicated significant differences in survival distributions across the 3 NPAR tertiles (*P* < .001 for both all-cause and CVD mortality).

### 3.5. Sensitivity analysis

**Figure 4.**
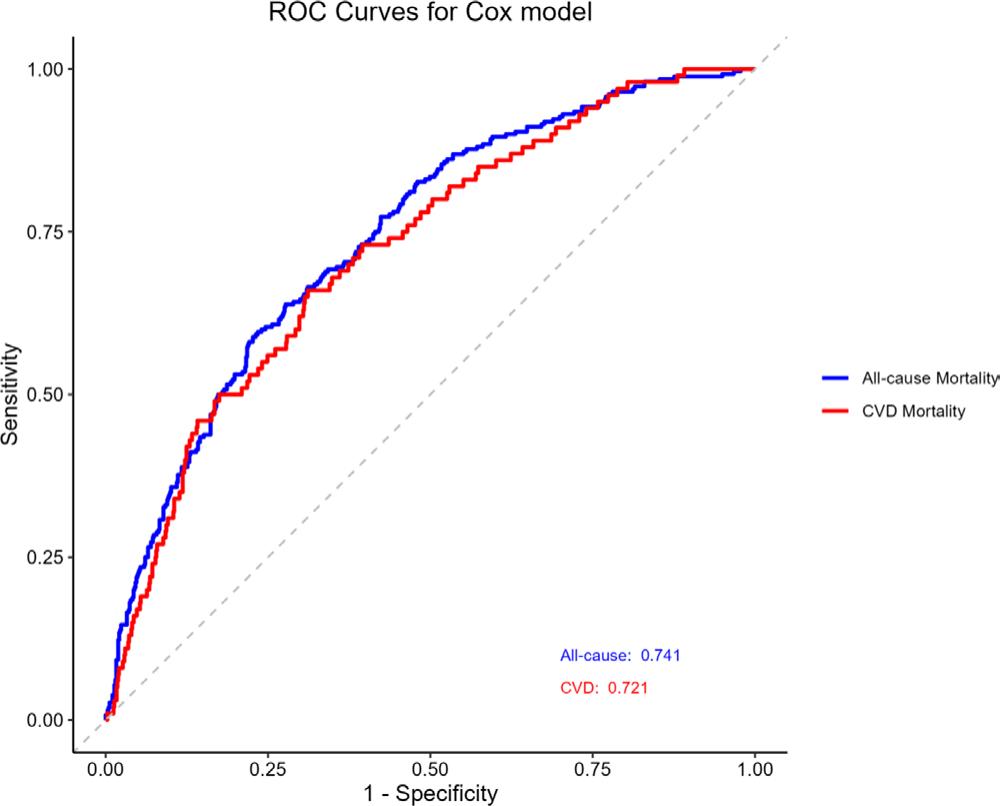
ROC curves for all-cause (blue) and CVD (red) mortality prediction. CVD = cardiovascular disease, ROC = receiver operating characteristic.

Receiver operating characteristic curves were generated for the fully adjusted Cox models (model 3) to assess their ability to discriminate between individuals who died and those who survived (Fig. 4). The area under the curve was 0.741 for predicting all-cause mortality and 0.721 for predicting CVD mortality, indicating acceptable discrimination performance for both models.

### 3.6. Subgroup analyses

**Figure 5.**
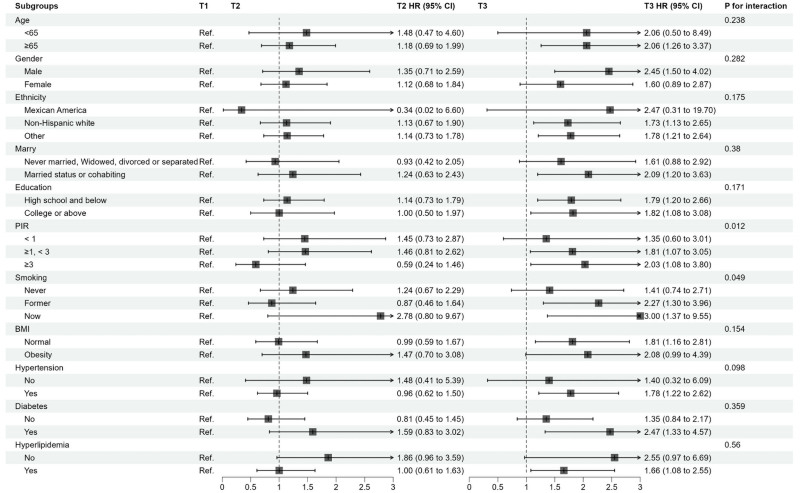
Subgroup analysis for all-cause mortality.

**Figure 6.**
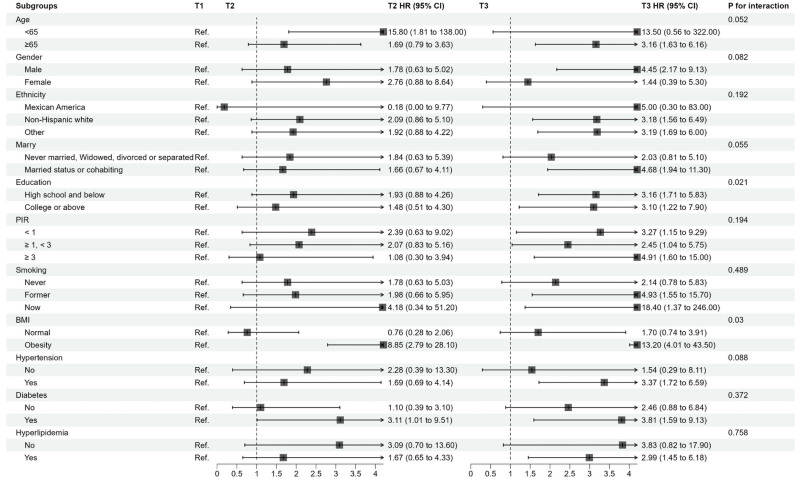
Subgroup analysis for cardiovascular disease mortality.

Subgroup analyses were conducted to explore potential effect modification of the association between NPAR (as a continuous variable) and mortality risk by various baseline characteristics (Figs. 5 and 6). For all-cause mortality (Fig. 5), significant interactions were observed for PIR (*P* for interaction = .012) and smoking status (*P* for interaction = .049). For CVD mortality (Fig. 6), significant interactions were found for BMI (*P* for interaction = .030) and education level (*P* for interaction = .021). Despite these interactions, the trend of increased mortality risk with higher NPAR levels was generally consistent across most subgroups, with particularly notable associations observed among individuals aged ≥65 years, males, and Non-Hispanic Whites.

## 4. Discussion

Our analysis, utilizing data from the NHANES database spanning 2009 to 2018, uncovered a significant nonlinear relationship between NPAR concentrations and both all-cause mortality and cardiovascular mortality among individuals with CHD. This association remained significant after adjusting for multiple potential confounders. These findings suggest that NPAR may serve as a potentially valuable and readily accessible indicator for predicting long-term mortality risk in patients with CHD.

Atherosclerosis, the pathological basis of CHD, is characterized by chronic inflammation. Inflammatory processes, including the infiltration of inflammatory cells like neutrophils, contribute to atheromatous plaque progression, destabilization, and rupture, potentially triggering acute myocardial infarction (AMI).^[13]^ Elevated levels of inflammatory markers often precede AMI, and a persistent inflammatory response typically accompanies the event.^[14]^ Neutrophils (N), as key players in the innate immune response and inflammation, can directly contribute to adverse cardiovascular outcomes when elevated. Previous studies have demonstrated that neutrophil counts following AMI are a strong, independent predictor of subsequent heart failure and mortality.^[15]^ Furthermore, neutrophil percentage (N%) has been correlated with overall mortality in various cardiovascular conditions, including atrial fibrillation,^[16]^ chronic heart failure,^[17]^ and cardiogenic shock.^[18]^

ALB, beyond being a marker of nutritional status, also reflects systemic inflammation. Lower serum albumin levels (hypoalbuminemia) have been established as a prognostic marker not only in the general population but also across a spectrum of diseases, including cardiovascular conditions.^[19]^ The proposed underlying mechanisms for albumin’s protective role may involve its anti-inflammatory, antioxidant, anticoagulant, and antiplatelet aggregation properties.^[20]^ Hypoalbuminemia is particularly common in older patients with heart failure and can exacerbate the condition, significantly impacting survival rates.^[21]^ Several studies have indicated that serum albumin levels can predict the risk of adverse outcomes in patients with AMI or CVD, irrespective of the presence of overt hypoalbuminemia.^[22–24]^

NPAR uniquely combines these 2 readily available clinical indicators, reflecting both the inflammatory state (via N%) and nutritional/inflammatory status (via ALB). An increase in NPAR typically results from an elevated N% and/or decreased ALB. This composite marker has shown promise as a predictor of adverse clinical events in various diseases. For instance, a longitudinal analysis involving 798 patients with AMI demonstrated that admission NPAR level was an independent predictor of 180-day and 365-day all-cause mortality.^[25]^ Another study reported that elevated NPAR was associated with higher in-hospital mortality and reinfarction rates in patients with ST-elevation myocardial infarction.^[26]^ Consistent with these prior findings, our study observed that significantly higher NPAR values were associated with a markedly increased risk of both all-cause and CVD death in individuals with established CHD.

Furthermore, our analysis revealed significant differences in NPAR levels across subgroups defined by PIR, smoking status, BMI, and education level. This aligns with previous suggestions^[27]^ that unhealthy lifestyle factors (such as smoking and obesity) and metabolic dysregulation can contribute to systemic inflammation, consequently influencing both neutrophil and albumin levels, and ultimately affecting NPAR. The interaction analyses in our study further support the potential modifying roles of these factors. The precise mechanisms linking lower educational attainment to higher NPAR warrant further investigation, though socioeconomic factors influencing lifestyle and access to care likely play a role. Collectively, these findings underscore the importance of addressing unhealthy lifestyle factors in the clinical management of CHD patients.

Compared with previous clinical studies with small sample sizes and many confounding factors, this study used large-scale and highly representative NHANES database as the research basis, considered and controlled for many potential confounding factors and integrated multiple analytical methods to make the results more reliable. The indicator in this study, NPAR, with its easy and rapid access and monitoring, provides timely and accurate reference information for clinical decision, it serves as a valuable early warning tool for risk assessment.

However, our study also has some shortcomings. Firstly, we measured the concentrations of N and ALB only once, which prevented us from observing the dynamic trends of NPAR changes. Secondly, random measurement errors were unavoidable. Additionally, while we accounted for possible confounding variables, we couldn’t entirely rule out the influence of residual or unidentifiable confounders. Therefore, in future studies, we need to explore more comprehensive and systematic methods to control and minimize the effects of these confounding factors ensuring our findings are more accurate and repeatable .

## 5. Conclusion

In this investigation, we identified a significant, nonlinear association between higher NPAR levels and increased risks of all-cause and cardiovascular mortality in individuals with CHD. These findings suggest that NPAR, as a readily accessible biomarker, holds potential prognostic value and may warrant attention in the comprehensive risk assessment and management of patients with CHD.

## Acknowledgments

We extend our thanks to the staff and participants of the National Health and Nutrition Examination Survey (NHANES) for their valuable contributions.

## Author contributions

**Conceptualization:** Han Yan.

**Data curation:** Jiayi Chen, Haorui Zha.

**Formal analysis:** Jiayi Chen, Haorui Zha.

**Funding acquisition:** Linghua Pei.

**Methodology:** Han Yan.

**Project administration:** Haorui Zha.

**Software:** Jiayi Chen.

**Supervision:** Linghua Pei.

**Validation:** Linghua Pei.

**Visualization:** Haorui Zha.

**Writing – original draft:** Han Yan, Jiayi Chen.
